# Psychometric Properties of General Self-Efficacy (GSE) Scale Korean Version for Older Korean Immigrants with Diabetes: A Cross-Sectional Study in the United States

**DOI:** 10.3390/nursrep13020074

**Published:** 2023-05-29

**Authors:** Jung Eun Kim, Ying-Hong Jiang, Vivien Dee

**Affiliations:** 1 Mennonite College of Nursing, Illinois State University, Normal, IL 61790, USA; 2 School of Education, Azusa Pacific University, Azusa, CA 91702, USA; yjiang@apu.edu; 3 School of Nursing, Azusa Pacific University, Azusa, CA 91702, USA; vdee@apu.edu

**Keywords:** self efficacy, psychometric, diabetes mellitus, emigrants and immigrants, reliability, validity

## Abstract

Patients with diabetes must have self-efficacy to perform necessary self-care tasks. Self-efficacy has been considered as one of the primary motivators on diabetes self-care; therefore, it is essential for health care professionals to assess the self-efficacy of patients with diabetes to provide optimal care. Despite older Korean immigrants having greater difficulty in diabetes management, research on self-efficacy for them is lacking. This study aims to examine the psychometric property of the General Self-Efficacy scale Korean version for older Korean immigrants with diabetes in the United States. In this cross-sectional, methodological study, data were collected using convenience sampling. Cronbach’s alpha, exploratory factor analysis, and confirmatory factor analysis were employed to examine the psychometric properties. Cronbach’s alpha for the entire GSE scale Korean version is 0.81. The initial Eigenvalues show two factors, coping and confidence; however, the confirmatory factor analysis showed reasonable goodness of fit to the data (*χ*^2^(35) = 86.24, *p* < 0.01), *χ*^2^/*df* ratio = 2.46, AGFI = 0.87, GFI = 0.91, IFI = 0.90, ECVI = 0.74, CFI = 0.89, and RMSEA = 0.093 in the one-factor model. The General Self-Efficacy scale Korean version demonstrated acceptable reliability and validity. It can be used to investigate self-efficacy and to devise culturally tailored diabetes interventions.

## 1. Introduction

In 2021, diabetes affected approximately 537 million adults (20 to 79 years) worldwide, with type 2 diabetes (T2D) accounting for approximately 90% of all types of diabetes [1]. Particularly, diabetes is widely acknowledged as a prevalent chronic condition that significantly affects the morbidity and mortality of older people. Approximately 48.8% of adults 65 years or older (26.4 million) have prediabetes in the United States [2]. Due to aging-related barriers, older individuals with diabetes face more challenges in maintaining optimal health status. Even though self-care is the most essential part of the effective management of diabetes, many older adults have difficulty performing effective self-care, because performing daily self-care activities is not simple. It is complex and includes multiple tasks such as diet changes, medication taking, monitoring blood sugar, and regular medical visits [3]. If patients with diabetes do not carry out the required self-care tasks correctly, they may experience acute or long-term consequences, such as eye and skin complications, functional disorder, neuropathy, hypertensive disorder, stroke, and even mortality [4]. 

To initiate self-care activities, individuals with diabetes must have internal motivation. Self-efficacy has been considered one of the primary motivators to change behaviors [5]. Bandura first proposed the concept of self-efficacy [5]; it represents an individual’s belief in their capacity to perform particular practices or tasks. Self-efficacy influences the efforts individuals are willing to exert in the face of barriers, obstacles, or failures [6]. For individuals with chronic illnesses, self-efficacy is the belief that one can exert control over challenging circumstances [7]. In other words, people with high self-efficacy are more likely to possess the highest levels of behavioral change ability [8]. In terms of behavior change, diabetes requires behavior changes to healthy lifestyles, and individuals with diabetes should have sufficient self-efficacy. Much of the previous literature reported that self-efficacy is one of the most important factors in self-care in patients with diabetes [8,9,10]. Therefore, measuring and identifying levels of self-efficacy for people with chronic disease such as T2D are essential for health care professionals to provide customized, effective health care services. 

In 1981, Matthias Jerusalem and Ralf Schwarzer established the General Self-Efficacy (GSE) scale; the scale was created to assess a person’s general sense of self-efficacy and self-beliefs in coping with daily challenges and adapting to all types of stressful life events [11]. The original developers of the GSE scale granted permission to other researchers to reproduce or employ it in future studies. The GSE scale is available in 32 languages, including Korean, and the various language versions are posted on their website (http://www.ralfschwarzer.de/ accessed on 10 March 2023).

Even though the GSE scale has been utilized in numerous studies, the psychometric properties of the Korean version of the GSE scale in older Korean immigrants with diabetes residing in the United States have not been investigated. For future research on self-efficacy related to self-care for older Korean immigrants with diabetes, it is crucial to examine the reliability and validity of the GSE scale Korean version. The findings of this study can also be applied to future studies on self-care for other Korean populations. The GSE scale Korean version translated and validated by Lee et al. [12] was utilized in this study. The GSE scale measures the general level of self-efficacy to deal with day-to-day challenges and stressful life events. One of the questions is “If I try hard enough, I can always solve difficult problems”. The GSE scale consists of 10 items. Each item contains four possible responses: “not at all” (1 point), “barely true” (2 points), “moderately true” (3 points), and “exactly true” (4 points). The total score is the sum of all items. The total score ranges between 10 and 40, with higher scores indicating greater self-efficacy.

### 1.1. Background

Among the various immigrant groups in the United States, Korean immigrants are one of the ethnic minorities. According to Migration Information Source [13], 16% of the total Korean immigrant population is constituted of those over 65 years of age, which is slightly higher than the overall proportion of older Americans who are immigrants (14%) [13]. The majority of older Korean immigrants are monolingual, and more than 70 percent of them have trouble comprehending medical terms and utilizing translated informational materials [14]. In contrast to younger Korean immigrants, older Korean immigrants face greater difficulties in managing diabetes due to limited English literacy and limited access to health care services [15]. They are marginalized in access to insurance and adequate treatment [16], and they have more challenges in performing self-care activities to manage their diabetes. To activate daily self-care activities, they should have confidence or belief that they can accomplish required tasks. The belief to accomplish is called self-efficacy, and measuring self-efficacy is crucial for health care professionals to provide optimal care services. 

Self-efficacy measuring instruments are classified generically into general and specialized scale categories. Several instruments, including the Diabetes Management Self-Efficacy Scale (DMSES) by Lee et al. [17], evaluate self-efficacy in relation to particular behaviors or situations. Alternatively, some instruments view self-efficacy as a more general trait; the GSE scale defines self-efficacy as a person’s overall competence to perform across a variety of life issues [18]. Despite the fact that a number of studies [19,20] have confirmed that the GSE scale has a high level of construct validity, additional research is necessary for various populations.

### 1.2. Conceptual Framework

This study was guided by Orem’s self-care deficit nursing theory (SCDNT) [21]. The Orem’s SCDNT has been widely implemented in clinical practice [22,23,24]. Orem’s self-care framework includes six fundamental concepts: self-care, self-care agency, therapeutic self-care demand, self-care deficit, nursing agency, and nursing system [21]. One of the six concepts, self-care agency, refers to the capacity to perform self-care, and the key concept of this study, self-efficacy, is aligned with self-care agency. 

The aims of this study were to assess the reliability and validity of the Korean version of the General Self-Efficacy (GSE) scale and examine the relationships between self-efficacy and socio-demographic characteristics of older Korean immigrants with diabetes. 

## 2. Materials and Methods

### 2.1. Participants

This was a cross-sectional study. Participants were recruited from two Southern California congregations serving the Korean community and from social media websites. A convenience sampling strategy was used to select participants. There are various definitions of “older adults” because the aging process is not uniform across the population because of genetic, lifestyle, and health differences [25]. In this study, an older adult is defined as a person aged 55 or older, given that the California Department of Aging provides retirement community accommodation to adults aged 55 and older [26]. The eligible participants were as follows:

- Korean immigrants who are 55 years old or older and reside in the US;
- Diagnosed with diabetes;
- Able to read and write in Korean;
- Able to give consent to participate in the survey; and
- Complete all items of the survey.

This research was approved by the Institutional Review Board of University (IRB ID number: 20-342). Before participation, the primary purpose, benefits, risks, and confidentiality rights of this study were explained to all participants. There was no compensation to participate in the research.

### 2.2. Data Collection

This research included both a paper survey and an online survey. In Southern California, a paper survey was conducted at two Korean community-based congregations. The primary researcher obtained written permission from the congregations to conduct the paper survey. After obtaining permission from the sites, recruitment flyers were posted within the churches’ structures. The primary investigator evaluated the eligibility of participants. In the presence of the principal researcher, eligible participants could complete the paper survey and return it directly to the researcher on-site. The online survey was conducted using the SurveyMonkey online survey platform of Momentive Global Inc. in San Mateo, California, the United States. The hyperlink to the SurveyMonkey online survey was posted on social network websites including Instagram, Facebook, Twitter, and internet community websites for Korean immigrants. Interested individuals participated in the survey immediately through the online link, and they could share the link to encourage others to participate. The compilation of data occurred between 3 October 2020 and 30 June 2021.

### 2.3. Measures

In the questionnaire, the Korean version of the GSE scale [12] was used, along with a brief socio-demographics section containing queries about gender, age, marital status, living status, educational level, employment status, annual income, health insurance, religion, years of residency in the US, and diagnosis of diabetes. 

### 2.4. Data Analysis

Version 26 of the Statistical Package for the Social Sciences (SPSS) from IBM, Chicago, Illinois, the United States was utilized for data analysis. Initially, the characteristics of the participants were analyzed using descriptive statistics, percentages, and frequencies. Using means and standard deviations, the self-efficacy level was calculated. The General Self-Efficacy (GSE) scale’s psychometric properties were described using Cronbach’s alpha, exploratory factor analysis (EFA), and confirmatory factor analysis (CFA). The results were compared with the results of the psychometric properties to previously published studies. The relationships between self-efficacy and participant characteristics were evaluated using independent t-tests, one-way analysis of variance (ANOVA), and Pearson’s correlation coefficients.

## 3. Results

### 3.1. Participants’ Characteristics and Self-Efficacy

Participants’ characteristics are provided in Table 1. From the paper and online survey, 603 responses were collected. Due to the COVID-19 pandemic, the online survey received the majority of responses. On the online survey, there were numerous incomplete responses. *n* = 171 was the total number of participants who met all inclusion criteria after deletion of incomplete data. 

The percentage of female participants was marginally higher than that of male participants (51.5% versus 48.5%). Of the 171 participants, 84 (49.1%) were between the ages of 55 and 64, while 87 (50.9%) were at least 65 years old. The median age of participants was 67.3 (SD = 9.9; range, 55–93). The majority of participants were married (69.0%). 25 (14.6%) and 23 (13.5%) participants were widowed and divorced, respectively. Among the 171 participants, 129 (75.4%) lived with family or relatives, 38 (22.2%) lived alone, and three (1.8%) lived with non-family or friends. 

The majority of participants (73.1%) possessed a bachelor’s degree or higher. Only 13 (7.6%) participants lacked a high school diploma. Regarding employment, 54.4% of respondents were unemployed, while 45.6% were employed. In addition, the annual income of 74 (43.3%) participants was less than USD 30 k, while the annual income of 29 (26.1%) participants fell between USD 30 k and USD 50 k. A total of 68 participants (39.8%) reported an annual income in excess of USD 50 k. 

Among the 171 participants, 94 (55.0%) had Medicare or Medi-Cal coverage, while 17 (9.9%) did not. The preponderance of participants (84.2%) were Christian. The majority of participants (96.5%) had lived in the United States for more than 10 years, while only six (3.5%) had lived in the country for fewer than 10 years. 

Each of the ten items on the General Self-Efficacy (GSE) scale has a point value between 1 and 4. Higher scores indicate a stronger sense of self-efficacy. The mean total self-efficacy score in this study was 29.6 out of 40 (SD = 3.6, range 19–39). 

### 3.2. Exploratory Factor Analysis 

Initial Eigenvalues derived from exploratory factor analysis indicate that two factors (coping and confidence) explain 39.3% and 11.5% of the variance, respectively. The Varimax rotation method produced a solution containing two interpretable factors, namely coping and confidence. Six items (Q5, Q6, Q7, Q8, Q9, Q10) account for 32.3% of the item variance with factor loadings ranging from 0.56 to 0.80, whereas four items (Q1, Q2, Q3, Q4) account for 18.5% of the item variance with factor loadings ranging from 0.40 to 0.80. Overall, coping and confidence accounted for 50.8% of the variance in the variable (see Table 2 and Table 3).

### 3.3. Reliability of General Self-Efficacy Scale-Korean Version

To examine the internal consistency, Cronbach’s alpha was utilized. Cronbach’s alpha for the General Self-Efficacy scale as a whole is 0.81, while the alphas for the coping and confidence subscales are 0.83 and 0.54, respectively.

### 3.4. Confirmatory Factor Analysis 

The relationship between latent and observed variables of the Korean variant of the GSE scale was investigated using a confirmatory factor analysis (CFA). The LISREL^®^ program was utilized for covariance matrix-based data analysis employing maximum likelihood estimation [27]. 

According to Schwarzer and colleagues [28], the GSE scale revealed one universal CFA factor. Nonetheless, the Korean version of the GSE scale revealed a two-factor model in the EFA results of this study. Consequently, this study evaluated both models, including Model A’s one-factor model and Model B’s two-factor model. Through conducting the CFA, it was discovered that Model B, two-factor model, showed better goodness of fit to the data compared to the Model A’s one-factor model. See Table 4. 

However, according to the results of EFA, in the two-factor Model B, the subscale confidence’s reliability is 0.54, which is unacceptable. Therefore, a one-factor solution, Model A is recommended when using the Korean version of the GSE scale.

**Figure 1:**
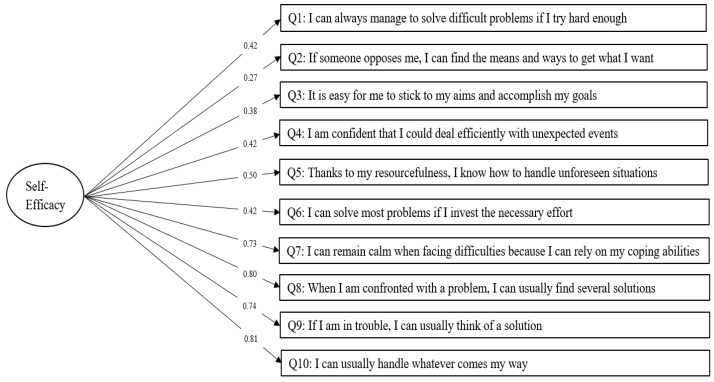
Model A: Confirmatory Factor Analysis with Standardized Solutions for a One-Factor Model with Ten Items on the General Self-Efficacy Scale, Korean Version.

The standardized solutions by CFA for the one-factor model, Model A are described in Figure 1.

### 3.5. Patient Characteristics and Self-Efficacy

According to the correlation matrix, the higher the self-efficacy of participants, the more likely they are to have a higher educational level (*r* = 0.186, *p* < 0.05), higher annual income (*r* = 0.170, *p* < 0.05), and longer residency in the U.S. (*r* = 0.248, *p* < 0.01). The older aged participants are a lower education level (*r* = −0.321, *p* < 0.01), lower annual income (*r* = −0.241, *p* < 0.01), and longer years in the U.S. (*r* = 0.239, *p* < 0.01). See Table 5. Participants who have a higher education level are more likely to have a higher annual income (*r* = 0.225, *p* < 0.01).

According to the results of *t*-tests and ANOVA, living arrangement (F(19,151) = [2.668], *p* < 0.001) and years of residency in the US (F(19,151) = [2.417], *p* = 0.002), the two characteristics revealed statistically significant differences on the total self-efficacy score. The other characteristics, including gender, age, educational level, employment, annual income, health insurance, and religion, did not show significant differences in self-efficacy scores. 

## 4. Discussion

The purpose of this study was to evaluate the psychometric properties of the Korean version of the GSE scale among older Korean immigrants with diabetes living in the United States. The results suggest that the GSE scale is legitimate and reliable for Korean immigrants with diabetes. This study reveals that Cronbach’s alpha coefficient for the overall measure is 0.81, indicating that the questionnaire was satisfactory. This study’s Cronbach’s alpha is comparable to 0.87 for the Thai version of the GSE among type 2 diabetes patients [29] and greater than 0.71 for the Brazil version among civil personnel [30]. The developers of GSE scale, Jerusalem and Schwarzer [11], discovered a Cronbach’s alpha of 0.75. Specifically, Luszczynska and Schwarzer [31] investigated the validity of the GSE scale in numerous nations. The reliability was 0.94 among German heart disease patients, 0.89 among German cancer patients, 0.90 among Polish students, 0.87 among Polish gastrointestinal disease patients, 0.87 among Polish swimmers, and 0.86 among South Korean participants.

Significantly, the EFA revealed two factors, coping and confidence; however, Cronbach’s alpha for the confidence subscale was 0.54, indicating that it was not reliable. Therefore, it is advised to use either the full GSE scale-Korean version or the subscale coping alone. 

The construct validity was evaluated using exploratory and confirmatory factor analysis in this study. In contrast to previous research [11,32], the EFA revealed that the GSE consisted of two dimensions, including confidence and coping. Nonetheless, in terms of the subscale confidence showing low Cronbach’s alpha, the GSE scale Korean version should be used as unidimensional. Scholz et al. [32] examined the psychometric properties of the GSE with 19,120 participants from 25 countries and demonstrated that the GSE is unidimensional. On the other hand, despite the fact that previous research has established that the GSE scale is a unidimensional and universal construct, many questions remain unanswered. Scholz et al. [32] found that Costa Ricans had the highest GSE level and Japanese had the lowest GSE sum score, indicating that the GSE sum score varied between nations. The structure of tools could also vary or change depending on the target population’s culture or lifestyle. Thus, future studies need to examine other populations. 

Regarding the differences in dimensions and sum score of the GSE among different nations, there could be several assumptions. First, it could be affected by different conditions of data collection. The circumstance of data collection could involve diverse uncontrolled variables to affect the results. Second, most previous studies used nonprobability sampling methods, which could be related to selection bias. Validating tools among different cultural groups is a never-ending process. 

Despite being statistically significant in the correlation of the GSE and the characteristics of participants, this study showed a weak to moderate association between the GSE and the characteristics of participants. However, the correlation highlights that the positive relationship between years in the US and self-efficacy may be meaningful. The longer Korean immigrants with diabetes reside in the US, the higher their self-efficacy levels are likely to be. In addition, the higher the annual income, the higher their self-efficacy level is likely to be. It may mean that economic status and length of residency in the US affect their self-efficacy level. Additionally, the implications of the statistically significant difference between living status and self-efficacy scores should be investigated in the future. 

There are no suggested cut-points for the GSE scale to distinguish between low and high self-efficacy. According to the original version, a cumulative score between 10 and 40 indicates greater self-efficacy. In this investigation, the average GSE score is 29.6 out of 40 (SD = 3.6, range 19–39), which is comparable to the 29.55 (SD = 5.32) obtained by Scholz et al. [32], who analyzed 19,120 individuals from 25 countries. In addition, the mean score of 29.6 is higher than that of Qiu et al. [33], who investigated the relationship between self-efficacy and diabetes knowledge among Chinese adult patients with diabetes. It is also greater than the 25.6 reported by Long et al. [34], who examined the role of self-efficacy as a mediator between perceived stress and quality of life among rural Chinese female patients with a history of gestational diabetes.

This study has a number of limitations. Initially, a paper questionnaire was intended to be used to collect a large sample for this research. Nonetheless, the COVID-19 pandemic occurred during the data collection phase, and the vast majority of data were collected through an online survey. Consequently, older respondents who had trouble accessing the internet or were unfamiliar with using a computer were unable to participate in the online survey. Therefore, the results cannot be generalized to other populations. In addition, the COVID-19 pandemic may have affected the participants’ perceptions of self-efficacy; however, the contextual issue was not investigated in this study. Future research must investigate the influence or causal effect of the COVID-19 pandemic on the perception of self-efficacy. This study investigated the associations between socio-demographic factors and self-efficacy. However, the relationships between self-efficacy and other constructs such as depression, anxiety, optimism were not investigated in this study. This is one of this study’s limitations. Moreover, with regard to the CFA results, this study demonstrated the greatest goodness of fit in the two-factor model, despite the fact that previous research suggested one factor solution. However, since the subscale of the two-factor model, confidence’s reliability, is not an acceptable value (0.54), it is recommended that the one-factor model be used. In the future, this result should be investigated. 

### New Contribution to Nursing Practice

Despite its limitations, this study has many positive qualities. This was the first study to evaluate the psychometric properties of the GSE scale among Korean diabetes immigrants in the United States, as far as we are aware. This study demonstrates that the Korean version of the GSE scale is psychometrically sound, reliable, and applicable for use with older Korean immigrants in the United States who have diabetes. The Korean version of the GSE scale can also be used to investigate how self-efficacy influences the health outcomes of diabetic patients. The Korean version of the GSE scale is probably valid for other chronic diseases, such as hypertension, in the older Korean immigrant population. 

Self-care is essential in the management of diabetes, and patients must be highly motivated to engage in essential self-care behaviors. The motivation is aligned with self-efficacy, and measuring self-efficacy among patients with diabetes is essentially required.

## 5. Conclusions

This study demonstrated the validity and reliability of the Korean version of the GSE scale for measuring General Self-Efficacy in older Korean immigrants with diabetes. In addition, the Korean version of the GSE can be used to investigate factors related to self-care among Korean immigrants with other chronic conditions. The findings of this study can aid in the creation of culturally sensitive interventions and the prevention of diabetes complications.

## Figures and Tables

**Table 1:** Participants’ Characteristics.

Variables	Response	*n =* 171	Percentage (%)
Gender	Male	83	48.5
	Female	88	51.5
Age (years)	55–59	42	24.6
	60–64	42	24.6
	65–69	30	17.5
	70–74	14	8.2
	75–79	14	8.2
	80–84	17	9.9
	More than 85 years	12	7.0
Marital status	Never married	1	0.6
	Married	118	69.0
	Separated	4	2.3
	Divorced	23	13.5
	Widowed	25	14.6
Living status	Living in facilities	1	0.6
	Living alone	38	22.2
	Living with family or relatives	129	75.4
	Living with non-family or friends	3	1.8
Educational level	Less than high school graduate	13	7.6
	High school graduate	33	19.3
	College or associate degree	44	25.7
	Bachelor’s degree or higher	81	47.4
Employment status	Employed	78	45.6
	Unemployed	93	54.4
Annual income	Less than 10 k	21	12.3
	10 k–19,999	41	24.0
	20 k–29,999	12	7.0
	30 k–39,999	18	10.5
	40 k–49,999	11	6.4
	50 k–59,999	16	9.4
	60 k–69,999	9	5.3
	70 k–79,999	9	5.3
	80 k–89,999	5	2.9
	90 k–99,999	4	2.3
	100 k or more	25	14.6
Health insurance	Medicare	45	26.3
	Medi-Cal	49	28.7
	Private insurance	60	35.1
	Uninsured	17	9.9
Religion	Christianity	144	84.2
	Buddhist	2	1.2
	Islam	0	0.0
	Hinduism	0	0.0
	Other	2	1.2
	None	23	13.4
Years of residency in the United States	Less than 10 years	6	3.5
	10–19	27	14.6
	20–29	53	28.7
	30–39	52	27.5
	40–49	39	19.9
	More than 50 years	12	5.8
Diagnosis of diabetes	Yes	171	100.0
	No	0	0

**Table 2:** Factor Loadings for Varimax Orthogonal and Two-Factor Solution for the Items of the GSE Scale.

Item	Factor Loadings	
	Factor 1	Factor 2
Factor 1: Coping (α = 0.83)		
Q10. I can usually handle whatever comes my way.	**0.80**	0.24
Q8. When I am confronted with a problem, I can usually find several solutions.	**0.78**	0.26
Q9. If I am in trouble, I can usually think of a solution.	**0.76**	0.17
Q7. I can remain calm when facing difficulties because I can rely on my coping abilities.	**0.69**	0.36
Q6. I can solve most problems if I invest the necessary effort.	**0.62**	−0.09
Q5. Thanks to my resourcefulness, I know how to handle unforeseen situations.	**0.56**	0.22
Factor 2: Confidence (α = 0.54)		
Q2. If someone opposes me, I can find the means and ways to get what I want.	−0.05	**0.80**
Q3. It is easy for me to stick to my aims and accomplish my goals.	0.17	**0.66**
Q4. I am confident that I could deal efficiently with unexpected events.	0.28	**0.53**
Q1. I can always manage to solve difficult problems if I try hard enough.	0.36	**0.40**

Note. *n* = 171 and Cronbach’s alpha for the entire measure is 0.81.

**Table 3:** Eigenvalues, Variance Percentages, and Cumulative Percentage for Factors in 10-Item GSE Scale.

Factor	Eigenvalue	% Variance	Cumulative %
1. Coping	3.93	32.3	32.3
2. Confidence	1.15	18.5	50.8

**Table 4:** Goodness-of-Fit Indices for Two Models of GSE Scale-Korean Version (*n =* 171).

Model	*df*	*χ* ^2^	*χ*^2^/*df Ratio*	AGFI	GFI	ECVI	CFI	IFI	RMSEA	90% CI
A	35	86.24	2.46	0.87	0.91	0.74	0.89	0.90	0.093	(0.068; 0.118)
B	34	77.57	2.28	0.88	0.93	0.70	0.91	0.91	0.087	(0.061; 0.112)

Note. *df* = degrees of freedoms; AGFI = adjusted goodness of fit; GFI = goodness of fit; ECVI = Expected Cross-Validation Index; CFI = Comparative Fit Index; IFI = Incremental Fit Index; RMSEA = Root Mean Square Error of Approximation; CI = Confidence Interval.

**Table 5:** All Variables’ Intercorrelations, Means, and Standard Deviations.

Variables	1	2	3	4	5	M	SD
1. Self-efficacy						29.56	3.60
2. Age	−0.126					67.29	9.95
3. Educational level	0.186 *	−0.321 **				3.13	0.98
4. Annual income	0.170 *	−0.241 **	0.225 **			52,817.62	88,774.76
5. Years in the U.S.	0.248 **	0.239 **	0.065	0.000		30.17	11.88

Note. * Correlation is significant at the *p* < 0.05 level (two tailed). ** Correlation is significant at the *p* < 0.01 level (two tailed). Bold values indicate statistical significance.

## Data Availability

The participants of this study did not give written consent for their data to be shared publicly, so due to the sensitive nature of the research, supporting data are not available.

## References

[1] International Diabetes Federation (2022). Diabetes Facts and Figures. https://www.idf.org/aboutdiabetes/what-is-diabetes/facts-figures.html.

[2] Centers for Disease Control and Prevention (2022). National Diabetes Statistics Report. https://www.cdc.gov/diabetes/data/statistics-report/.

[3] Weinger K., Beverly E.A., Smaldone A. (2015). Diabetes self-care and the older adult. West. J. Nurs. Res..

[4] American Diabetes Association (2023). Diabetes Overview. https://diabetes.org/diabetes.

[5] Bandura A. (1977). Self-efficacy: Toward a unifying theory of behavioral change. Psychol. Rev..

[6] Bandura A., Vasta R. (1989). Social cognitive theory. Annals of Child Development, Six Theories of Child Development.

[7] Schnell K.N., Naimark B.J., McClement S.E. (2006). Influential factors for self-care in ambulatory care heart failure patients: A qualitative perspective. Can. J. Cardiovasc. Nurs..

[8] Dehghan H., Charkazi A., Kouchaki G.M., Zadeh B.P., Dehghan B.A., Matlabi M., Mansourian M., Qorbani M., Safari O., Pashaei T. (2017). General self-efficacy and diabetes management self-efficacy of diabetic patients referred to diabetes clinic of Aq Qala, North of Iran. J. Diabetes Metab. Disord..

[9] Bohanny W., Wu S.-F.V., Liu C.Y., Yeh S.H., Tsay S.L., Wang T.J. (2013). Health literacy, self-efficacy, and self-care behaviors in patients with type 2 diabetes mellitus. J. Am. Assoc. Nurse Pract..

[10] Karimy M., Araban M., Zareban I., Taher M., Abedi A. (2016). Determinants of Adherence to Self-Care Behavior among Women with Type 2 Diabetes: An Explanation Based on Health Belief Model. http://mjiri.iums.ac.ir.

[11] Jerusalem M., Schwarzer R., Schwarzer R. (1992). Self-efficacy as a resource factor in stress appraisal processes. Self-Efficacy: Thought Control of Action.

[12] Lee Y., Schwarzer R., Jerusalem M. (1994). Korean Adaptation of the General Self-Efficacy Scale. http://userpage.fu-berlin.de/~health/korean.htm.

[13] Migration Information Source (2014). Korean Immigrants in the United States. https://www.migrationpolicy.org/article/korean-immigrants-united-states-2013.

[14] Han H.-R., Kim J., Kim M.T., Kim K.B. (2011). Measuring health literacy among immigrants with a phonetic primary language: A case of Korean American women. J. Immigr. Minor. Health.

[15] Song Y., Song H.-J., Han H.-R., Park S.-Y., Nam S., Kim M.T. (2012). Unmet needs for social support and effects on diabetes self-care activities in Korean Americans with type 2 diabetes. Diabetes Educ..

[16] Choi S., Rush E. (2012). Effect of a short-duration, culturally tailored, community-based diabetes self-management intervention for Korean immigrants. Diabetes Educ..

[17] Lee E.H., van der Bijl J., Shortridge-Baggett L.M., Han S.J., Moon S.H. (2015). Psychometric properties of the diabetes management self-efficacy scale in Korean patients with type 2 diabetes. Int. J. Endocrinol..

[18] Zhang J.X., Schwarzer R. (1995). Measuring optimistic self-belief: A Chinese adaptation of the general self-efficacy scale. Psychologia.

[19] Leganger A., Kraft P., R⊘ysamb E. (2000). Perceived self-efficacy in health behaviour research: Conceptualisation, measurement and correlates. Psychol. Health.

[20] Schwarzer R., Mueller J., Greenglass E. (1999). Assessment of perceived general self-efficacy on the Internet: Data collection in cyberspace. Anxiety Stress Coping.

[21] Orem D.E. (1991). Nursing: Concepts of Practice.

[22] Afrasiabifar A., Mehri Z., Sadat S.J., Shirazi H.R.G. (2016). The effect of Orem’s self-care model on fatigue in patients with multiple sclerosis: A single blind randomized clinical trial study. Iran. Red Crescent Med. J..

[23] Mahmoudzadeh Zarandi F., Raiesifar A., Ebadi A. (2016). The effect of Orem’s self-care model on quality of life in patients with migraine: A randomized clinical trial. Acta Med. Iran..

[24] Yip J.Y.C. (2021). Theory-based advanced nursing practice: A practice update on the application of Orem’s self-care deficit nursing theory. SAGE Open Nurs..

[25] Levine M.E. (2013). Modeling the rate of senescence: Can estimated biological age predict mortality more accurately than chronological age?. J. Gerontol. Ser. A Biol. Sci. Med. Sci..

[26] California Department of Aging (2023). Determine the Best Housing Option for Me. https://aging.ca.gov/Care_Options/Determine_the_Best_Housing_Option_For_Me/.

[27] Jőreskog K.G., Sőrbom D. (2018). LISREL 10 for Windows (10.3.3.26).

[28] Schwarzer R., Born A., Iwawaki S., Lee Y.M. (1997). The assessment of optimistic self-beliefs: Comparison of the Chinese, Indonesian, Japanese, and Korean versions of the General Self-Efficacy scale. Psychologia.

[29] Hurst C., Rakkapao N., Malacova E., Mongkolsomlit S., Pongsachareonnont P., Rangsin R., Promsiripaiboon Y., Hartel G. (2022). Psychometric properties of the general self-efficacy scale among Thais with type 2 diabetes: A multicenter study. PeerJ.

[30] Machado L.A.C., Telles R.W., Costa-Silva L., Barreto S.M. (2016). Psychometric properties of multidimensional health locus of control—A and general self-efficacy scale in civil servants: ELSA-Brasil musculoskeletal study (ELSA-Brasil MSK). Braz. J. Phys. Ther..

[31] Luszczynska A., Schwarzer U. (2005). The general self-efficacy scale: Multicultural validation studies. J. Psychol..

[32] Scholz U., Gutiérrez Doña B., Sud S., Schwarzer R. (2002). Is general self-efficacy a universal construct. Eur. J. Psychol. Assess..

[33] Qiu T., Huang J., Wang W. (2020). Association between diabetes knowledge and self-efficacy in patients with type 2 diabetes mellitus in China: A cross-sectional study. Int. J. Endocrinol..

[34] Long Q., Guo J., Zhong Q., Jiang S., Wiley J., Chen J.L. (2021). General self-efficacy and social support as mediators of the association between perceived stress and quality of life among rural women with previous gestational diabetes mellitus. J. Clin. Nurs..

